# Keroselene as an Anæsthetic

**Published:** 1862-10

**Authors:** 


Kerosolene as an Anæsthetic.—Considerable has been said, during the last twelve months, in regard to kerosolene as an anæsthetic, and many experiments made by different observers, with varying results. So far as these experiments have come to our observation, we have summed up the results and conclusions, for the benefit of our numerous readers. In the *American Medical Times* for February 8th, Dr. Asa Horr, of Dubuque, Iowa, has a paper upon this subject. He experimented upon himself first, then upon his son and wife, and finally, upon his patients. In all cases, he reports, the result is quite satisfactory. In his own case, "the effects produced were nearly similar to those I had often experienced from chloroform, but with a greater feeling of buoyancy, and less thrilling noise in the head, leaving no nausea nor giddiness."

Breathing the agent vigorously, the effects began in thirty seconds, increased (though the inhalation was discontinued), during fifty seconds, and then gradually declined. In two minutes the anæsthetic had wholly passed away.

Dr. Horr has administered the kerosolene immediately after a full meal, without the production of vomiting, as usually results when chloroform is administered under such circumstances. The usual unpleasant head symptoms were also absent after the anæsthesia had passed away.

He administered the remedy in a case of labor, of which he says:

"Prior to the inhalation she was restless, and greatly alarmed, but after a few inspirations of the anæsthetic, she became calm, and remained tranquil until delivery was accomplished, involving the forcible separation of the placenta. In half an hour she was wholly recovered from the anæsthetic, and expressed a decided preference for kerosolene over *ether*, which had been given during a regular labor three years before, and was used in large amount, attended with great mental and muscular excitement. She made a rapid and complete recovery."

Dr. Horr has also given keroselene, combined with chloroform, equal parts of each. Of the compound, he says:

"The use of this compound appears to be attended with less disturbance of the stomach, fuller circulation in the capillaries, and less irritation of the air-passages during inhalation than chloroform; otherwise, its action is precisely as if no kerosolene were added. The amount, by measure, to produce a given effect is but slightly greater than would be required of chloroform."

Dr. Horr has evidently been more fortunate than many other experimenters with the agent under consideration. We are still of the opinion that it will be found inferior either to chloroform or ether.—*Med. and Surg. Reporter.*

